# Croup: Its Nature and Homœopathic Treatment

**Published:** 1887-04

**Authors:** 


### BOOK NOTICES AND REVIEWS.

CROUP: ITS NATURE AND HOMŒOPATHIC TREATMENT, WITH ILLUSTRATIONS OF HOMŒOPATHIC PRACTICE. By Hurro Nauth Roy, L. M. S.: Lahiri & Co., Homœopathic Chemists, Calcutta, India, 1886.

This little pamphlet of forty-six pages is particularly interesting because it is written by a native East Indian physician. It gives a very excellent summary of the essential features of croup according to the latest information, and then states the remedies according to their therapeutic value. Clinical cases are given collected from various contributors to homœopathic literature. Two or three are added that are peculiar to India. They are as follows: Blatta Orient (cockroach) indicated in great dyspnœa, face congested, restlessness, hoarse, croupy cough, constantly changing position. Fever may or may not be present. Moocta Jhoree (Acalypha Indica), Toolsee (Ocymum Villosum), Beetel Leaf (Piper Chavica), and Kala of the natural order Acanthaceæ.

From the tenor of a quotation from Dr. Holcombe we infer that the sympathies of the author are with the liberal or eclectic wing of our school.

